# Wnt Signaling Deregulation in the Aging and Alzheimer’s Brain

**DOI:** 10.3389/fncel.2019.00227

**Published:** 2019-05-22

**Authors:** Ernest Palomer, Johanna Buechler, Patricia C. Salinas

**Affiliations:** Department of Cell and Developmental Biology, University College London, London, United Kingdom

**Keywords:** Wnt, aging brain, synapse degeneration, synaptic maintenance, Alzheimer’s disease

## Abstract

Growing evidence suggests that synaptic signaling is compromised in the aging brain and in Alzheimer’s disease (AD), contributing to synaptic decline. Wnt signaling is a prominent pathway at the synapse and is required for synaptic plasticity and maintenance in the adult brain. In this review, we summarize the current knowledge on deregulation of Wnt signaling in the context of aging and AD. Emerging studies suggest that enhancing Wnt signaling could boost synaptic function during aging, and ameliorate synaptic pathology in AD. Although further research is needed to determine the precise contribution of deficient Wnt signaling to AD pathogenesis, targeting Wnt signaling components may provide novel therapeutic avenues for synapse protection or restoration in the brain.

## Introduction

Wnt signaling was originally discovered as a tumorigenic pathway in the early 1980s. Almost four decades later, this prominent pathway has been implicated in numerous cellular processes from early embryonic development to tissue homeostasis. This signaling cascade is also linked to a variety of diseases, from cancer to bone and cardiovascular diseases. In the nervous system, Wnt signaling plays a vital role from early patterning of the nervous system to higher functions including synaptic plasticity and memory in the adult brain. In this review, we will focus on new studies indicating a deregulation of the Wnt signaling pathway in the aging brain and Alzheimer’s disease (AD).

### Alzheimer’s Disease

Alzheimer’s disease is a devastating neurodegenerative disorder that accounts for two thirds of all dementia cases, and is characterized by progressive cognitive impairment and memory loss leading to difficulties in the performance of daily tasks (Apostolova, 2016). The AD brain displays extracellular Amyloid beta (Aß) deposition, intracellular Tau aggregates (neurofibrillary tangles), and widespread neuronal death at late AD stages. However, synapse loss is the prominent event in early stages of AD and represents the best pathological correlate of cognitive decline in patients (Terry et al., 1991). Current models propose that synaptic dysfunction and degeneration in AD are initiated by oligomers of Aß, a key pathogenic molecule, which negatively regulates signaling pathways that are crucial for synaptic function and stability (Forner et al., 2017). Consistent with this view, increasing evidence suggests that Aß triggers deregulation of Wnt signaling, resulting in the dampening of this important cascade at the synapse. These changes in the Wnt pathway could contribute to synapse dysfunction and degeneration, thereby promoting the progression of AD.

### Wnt Signaling Pathway

**FIGURE 1:**
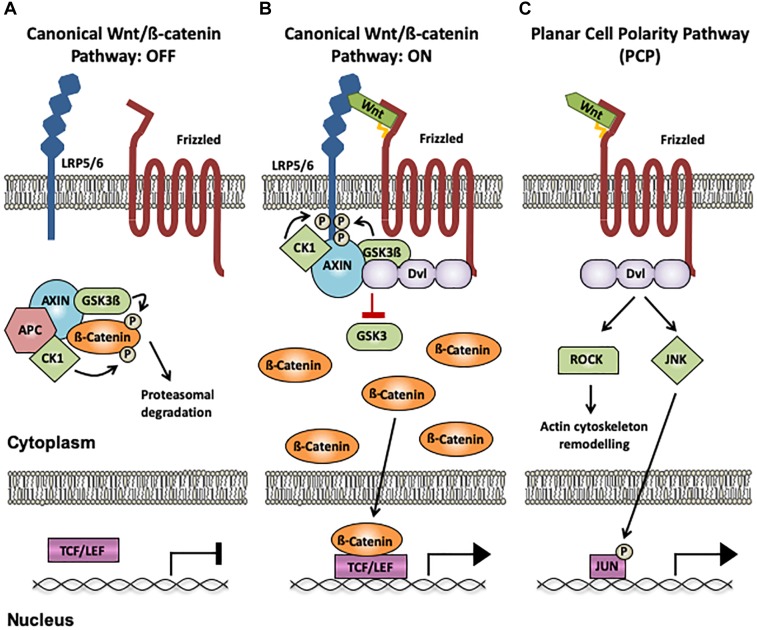
Wnt signaling pathways. **(A)** Canonical Wnt/ß-catenin pathway OFF: In the absence of Wnt binding to Frizzled (Fz) receptors and LRP5/6 co-receptors, ß-catenin interacts with the destruction complex (CK1, GSK3β, Axin1 and APC), resulting in its phosphorylation by Gsk3ß and CK1 and its subsequent degradation by the proteasome. **(B)** Canonical Wnt/ß-catenin pathway ON: When a Wnt ligand binds to LRP5/6 and Fz receptors, the scaffold protein Disheveled (Dvl) recruits Axin1 and the kinases CK1 and GSK3ß to the membrane, disrupting the destruction complex and impairing ß-catenin phosphorylation and degradation. ß-catenin accumulates in the cytoplasm and subsequently translocates to the nucleus, where it acts as an activator of TCF/LEF-mediated transcription of Wnt target genes. **(C)** The Planar Cell Polarity (PCP) pathway: The PCP does not require LRP5/6 and is ß-catenin-independent. When Wnts bind to Fz receptors, Dvl is activated, resulting in increased activation of ROCK and JNK through the small GTPases RhoA and Rac1, respectively. Changes in ROCK activity induce actin remodeling, and JNK activation can promote gene transcription via Jun phosphorylation. The palmitate group in Wnt ligands is indicated as a yellow tail. CK1, casein kinase 1; GSK3ß, glycogen synthase kinase 3β; Axin1, axis inhibition protein 1; APC, adenomatous polyposis coli; TCF, T-cell factor; LEF, lymphocyte-enhancer-binding factor; Rock, Rho associated coiled-coil-containing protein kinase 1; RhoA, RAS homolog gene-family member A; JNK, Jun N-terminal kinase.

Wnt proteins are a family of secreted lipoproteins that activate different intracellular signaling pathways by binding to several receptors and co-receptors at the cell surface. Three major Wnt signaling pathways have been described: canonical Wnt/ß-catenin, planar cell polarity (PCP), and Wnt/Ca^2+^ pathways, which lead to changes in gene expression and/or cytoskeleton reorganization. The most extensively studied cascade is the canonical Wnt pathway that controls the expression of Wnt target genes by stabilizing cytoplasmic ß-catenin and by inducing the reorganization of the cellular cytoskeleton (Nusse and Clevers, 2017). In absence of Wnt ligands, ß-catenin is phosphorylated by Gsk3ß and CK1 in the cytoplasm, leading to its degradation (Figure 1A; Nusse and Clevers, 2017). Upon binding of Wnt ligands to their receptors Frizzled and co-receptors LRP5/6, the scaffold proteins Disheveled (Dvl) and Axin are recruited to form a signalosome at the cell membrane. This in turn allows ß-catenin accumulation in the cytoplasm and its translocation to the nucleus, where it associates with TCF/LEF and other co-factors to regulate Wnt target gene transcription (Figure 1B; Nusse and Clevers, 2017). The Wnt PCP pathway leads to transcriptional changes and cytoskeleton reorganization upon Wnt ligand binding to Frizzled receptors, without the requirement of Wnt co-receptors LRP5/6 (Seifert and Mlodzik, 2007). This signaling cascade activates the small GTPases RhoA and Rac1, which in turn activate the kinases ROCK and JNK, respectively (Figure 1C; Seifert and Mlodzik, 2007). The third pathway is the Wnt/Ca^2+^ cascade, in which binding of Wnts to Frizzled receptors leads to phospholipase C (PLC) activation, release of Ca^2+^ from intracellular stores and activation of CaMKII and PKC, resulting in transcriptional changes and actin remodeling (De, 2011).

### Wnt Signaling at the Mature Synapse

Wnt signaling has been implicated in axonal pathfinding, dendritogenesis, synapse formation, synaptic plasticity, and maintenance (Budnik and Salinas, 2011; Dickins and Salinas, 2013; McLeod and Salinas, 2018). A fine balance of canonical and non-canonical PCP Wnt signaling is required for maintaining mature synapses. Decreased levels of canonical Wnt signaling with the concomitant activation of the PCP pathway leads to synapse disassembly (Marzo et al., 2016; Sellers et al., 2018). In addition, canonical and non-canonical Wnt pathways play a role in synaptic plasticity at the mature synapse. For example, different Wnt proteins are involved in early or late phases of long-term potentiation (LTP). *In vivo* studies show that canonical Wnt signaling is required for the early phase of LTP and also sufficient to enhance it (Ivanova et al., 2017). Non-canonical Wnt signaling pathways have been implicated in both early and late phases of LTP. Wnt7a/b through its receptor frizzled 7, participates in early LTP phases by promoting AMPAR recruitment at the synapses *in vitro* (McLeod et al., 2018). As this process is CaMKII dependent, it is likely to be due to the activation of Wnt/Ca^2+^ pathway. In addition, Wnt5a regulates basal NMDAR currents and influences late phase of LTP by activating the Wnt PCP pathway in hippocampal slices (Cerpa et al., 2011). Altogether, these studies demonstrate that canonical and non-canonical Wnt signaling are relevant pathways at the mature synapse.

## WNT Signaling and Aging

Reduced synaptic strength and function occur during normal aging (Morrison and Baxter, 2012; Petralia et al., 2014). Age is the biggest risk factor for late-onset AD, as growing synaptic vulnerability may increase the susceptibility of synapses to toxic molecules such as Aß. Consistent with this view, various signaling pathways, which are crucial for synapse integrity, undergo changes in the aging brain (Bishop et al., 2010). Of particular interest is the Wnt signaling pathway, which is required for synaptic plasticity (McLeod et al., 2018), synaptic maintenance (Marzo et al., 2016) and is altered during aging (Matarin et al., 2015; García-Velázquez and Arias, 2017).

### Wnt Signaling Deregulation in the Human Aging Brain

A recent study showed that Wnt components expression is affected in the aging human brain. Indeed expression of Wnt ligands *WNT2B*, *WNT6*, and *WNT7a* and frizzled receptors *FZD2* and *FZD3* is downregulated in the aged human brain (Folke et al., 2018). Additionally, the same study showed that the secreted frizzled-related protein 1 (*SFRP1*) is upregulated in the aged human brain (Folke et al., 2018). Interestingly, SFRPs sequester Wnt ligands in the extracellular space, thus increased *SFRP1* could interfere with many Wnt pathways. Together, these findings suggest that Wnt signaling is dampened in the aged human brain.

### Dampening Wnt Signaling in the Aged Rodent Brain

**FIGURE 2:**
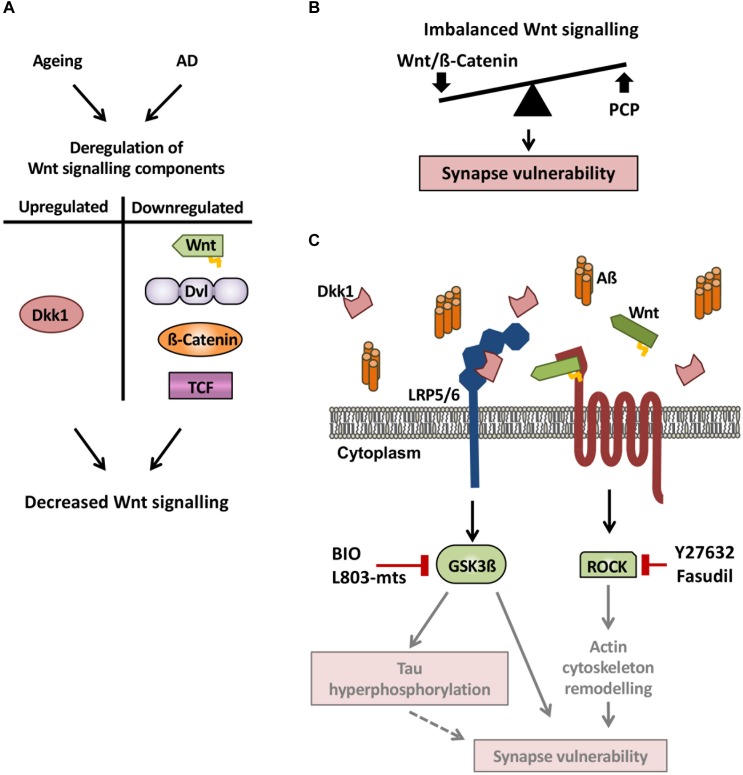
Wnt signaling deregulation in the aging brain and in AD. **(A)** Wnt signaling components are deregulated in both the aging and AD brain. Notably, the Wnt antagonist Dkk1 is upregulated, whereas Wnt ligands, Dvl, ß-catenin, and TCF are downregulated, leading to decreased canonical Wnt signaling. **(B)** As Dkk1 mainly affects the canonical Wnt signaling pathway, we propose a model by which elevation of Dkk1 results in an imbalance between the Wnt/ß-catenin and PCP pathways, resulting in synaptic defects and synapse loss. **(C)** In the brain of AD subjects and AD mouse models, the Wnt antagonist Dkk1 is elevated, leading to increased activity of both Gsk3ß and ROCK, thus resulting in reduced Wnt/ß-catenin signaling and increased PCP signaling. The activation of these two kinases leads to synapse vulnerability but the precise mechanisms downstream remain to be defined. A possible mechanism is that tau phosphorylation and actin remodeling contribute to synaptic changes (gray arrows and diffuse red boxes in the scheme) and subsequent cognitive deficits. These results suggest that pharmacologically targeting Wnt signaling could be a promising therapeutic strategy for synapse protection in AD.

In the aged rodent brain, several Wnt pathway elements are also downregulated. For example, the expression of the Wnt ligands *Wnt2*, *Wnt4*, and *Wnt9a*, and transcription factors *Lef1* and *Tcf3* is downregulated (Figure 2A; Hofmann et al., 2014). In the dentate gyrus region of the hippocampus, *Wnt3* and *Wnt3a* expression progressively declines between 1 and 22 months of age (Okamoto et al., 2011). Moreover, a specific reduction in canonical Wnt signaling is evident in the hippocampus of aged rats, where Dvl2, Axin2 and nuclear ß-catenin are downregulated (Figure 2A; Orellana et al., 2015). A similar deficiency in Wnt signaling is observed in a mouse model of accelerated aging (Bayod et al., 2015). Reduced Wnt signaling in the aging brain also arises from increased levels of endogenous secreted Wnt antagonist such as Dickkopf-1 (Dkk1) in the aging mouse brain (Figure 2A; Scott et al., 2013; Seib et al., 2013). Altogether these data demonstrate a dampening of Wnt signaling in the aging brain of rodents as not only Wnt ligands and intracellular components of the pathway are downregulated, but also the potent canonical Wnt antagonist Dkk1 is upregulated.

### Discrepancies Between the Human and Rodent Aging Brain

Although Wnt signaling is reduced in both the human and rodent aging brain, the specific deregulated Wnt signaling components are not exactly the same (see above). A possible explanation is that the studies have been conducted in different areas of the brain, where Wnt components are differentially expressed under basal conditions. For example, most of rodent studies analyzed the hippocampus, whereas human studies have been performed in the prefrontal cortex. Nonetheless, the data presented showed that Wnt signaling is reduced in the aging brain, possibly contributing to the observed age-related synaptic decline (Morrison and Baxter, 2012; Petralia et al., 2014). Thus, boosting Wnt signaling in the aging brain may strengthen synaptic resilience, slow down cognitive decline and decrease the risk of pathological transitions toward dementia in elderly individuals.

## WNT Signaling and Alzheimer’s Disease

Deregulation of Wnt signaling may also contribute to synapse vulnerability in the context of AD. Indeed, reduced Wnt signaling has a direct impact on synapses (Marzo et al., 2016). In addition, deficient Wnt signaling could also induce indirect effects on synapses by activating the amyloidogenic pathway (Liu et al., 2014) or by interfering with microglial survival (Zheng et al., 2017).

### Antagonizing Canonical Wnt Signaling in AD

Several studies have shown that the canonical Wnt antagonist Dkk1 is up-regulated in brains of AD patients and AD mouse models (Caricasole et al., 2004; Rosi et al., 2010). Dkk1 inhibits canonical Wnt signaling by interacting with LRP5/6 Wnt co-receptors, thus impairing the binding of Wnt proteins to both Frizzled and LRP5/6. This inhibition of Wnt signaling by Dkk1 leads to increased Gsk3ß activity and reduced cytoplasmic ß-catenin levels – both features which are observed in the brains of AD patients (Zhang et al., 1998; Pei et al., 1999; Kawamura et al., 2001). In addition, *Dkk1* expression is rapidly elevated by Aß in hippocampal neurons, resulting in reduced canonical Wnt signaling and concomitant synapse loss (Figure 2; Purro et al., 2012; Sellers et al., 2018). Interestingly, synapses are protected from Aß insult when Dkk1 is blocked, suggesting that Dkk1 is required for Aß-mediated synapse loss (Purro et al., 2012). To mimic the effect of Aß on synapses through Dkk1, a transgenic mouse model was generated that inducibly expresses Dkk1 in the brain (iDkk1 mice). Induced Dkk1 expression results in synapse loss in the striatum (Galli et al., 2014) and hippocampus (Marzo et al., 2016) without affecting cell viability. Moreover, iDkk1 mice exhibit reduced synaptic transmission, impaired LTP and enhanced LTD in the adult hippocampus, accompanied by long-term memory deficits (Marzo et al., 2016). Thus, increased expression of Dkk1 in the adult brain reproduces several of the phenotypic aspects observed in AD models (Marchetti and Marie, 2011; Esquerda-Canals et al., 2017). Interestingly, a variant of the Wnt/Dkk1 receptor LRP6, which displays reduced Wnt signaling in cell lines, is associated with an increased risk for late-onset AD (De Ferrari et al., 2007). This finding further strengthens the link between deficient Wnt signaling and AD. In addition, neuronal deletion of LRP6 in the postnatal forebrain leads to synaptic loss and exacerbates AD pathology in an AD mouse model (Liu et al., 2014). Collectively, these results support the view that Aß-induced inhibition of Wnt signaling through Dkk1 contributes to synaptic impairment and cognitive deficits in AD.

### Wnt Signaling Involvement in Aß Production and Tau Tangles

Several additional lines of evidence further suggest that deficiency in Wnt signaling contributes to AD pathogenesis. First, the canonical co-receptor LRP6 modulates the processing of the Aß precursor protein (APP) and knock-out of LRP6 leads to increased Aß production in an AD mouse model (Liu et al., 2014). This is further supported by *in vitro* studies showing that suppression of canonical Wnt signaling or activation of the Wnt-PCP pathway promotes the amyloidogenic processing of APP, leading to increased production of Aß through a feedback loop mechanism (Tapia-Rojas et al., 2016; Elliott et al., 2018). In addition, several other Wnt pathway components, including ß-catenin, Tcf4, Gsk3ß, and Dvl1, have been implicated in modulating APP processing (Mudher et al., 2001; Liu et al., 2014; Parr et al., 2015; Tapia-Rojas et al., 2016). Second, reduced Wnt signaling leads to increased Gsk3ß activity, which contributes to tau hyperphosphorylation (Scali et al., 2006; Salcedo-Tello et al., 2014), a key pathological hallmark of AD. Conditional overexpression of Gsk3ß in the brain causes neurodegeneration and learning deficits (Hernández et al., 2002). Collectively, this data suggests that reduced Wnt signaling is involved in a negative feedback loop promoting the exacerbation of AD pathology, which in turn would dampen Wnt signaling further by Aß-mediated increase in Dkk1 expression.

### AD Associated Genes Are Linked to Reduced Wnt Signaling

Several AD susceptibility genes (including *APOE*, *TREM2*, and *Clusterin*) are linked to aberrant Wnt signaling. For example, ApoE4, a major genetic risk factor for late-onset AD, inhibits canonical Wnt signaling in cell lines (Caruso et al., 2006). More recently, a study showed that TREM2, which is linked to late-onset AD, promotes microglia proliferation through Wnt signaling (Zheng et al., 2017). Finally, the AD-associated protein Clusterin (Harold et al., 2009; Lambert et al., 2009) is involved in Aß-driven *Dkk1* expression, as soluble Aß promotes the intracellular accumulation of Clusterin and subsequent Dkk1 upregulation, whereas knock-down of Clusterin prevents induction of *Dkk1* expression and protects against Aß neurotoxicity (Killick et al., 2014). Not only AD risk factors are linked to reduced Wnt signaling. For example, Wnt signaling promotes the expression of RE1-Silencing Transcription factor (REST) during normal aging (Willert et al., 2002; Lu et al., 2014). In turn, REST represses pro-apoptotic genes and exerts a protective function against oxidative stress and Aß neurotoxicity (Lu et al., 2014). Thus, diminished Wnt signaling could contribute to reduced REST levels observed in AD (Lu et al., 2014), resulting in increased susceptibility to Aß toxicity.

In summary, a growing body of evidence suggests that Wnt signaling is deregulated in AD, which could contribute to synapse degeneration and cognitive decline. This deficiency in Wnt signaling may further exacerbate key pathological processes including Aß production and Tau hyperphosphorylation.

## Protective Effects Of WNT Signaling Against Aß

Activation or restoration of Wnt signaling has protective effects in the context of Aβ-induced synaptic degeneration. Dkk1-induced synapse loss, impaired synaptic plasticity and cognitive defects can be fully reversed *in vivo* upon restoration of Wnt signaling through the cessation of Dkk1 expression (Marzo et al., 2016). *In vitro*, synapses can be protected against Dkk1 by inhibiting two kinases regulated by Wnt signaling: Gsk3ß and ROCK (Figure 2C; Marzo et al., 2016). Consistently, *in vivo* inhibition of Gsk3ß by the specific substrate-competitive inhibitor L803-mts peptide improves cognition in AD mouse models (Avrahami et al., 2013; Licht-Murava et al., 2016). In addition, *in vivo* inhibition of ROCK by fasudil improves cognition (Sellers et al., 2018) and reduces Aß load in AD rodent models (Elliott et al., 2018; Figure 2C). These studies suggest that Dkk1-induced synapse loss is reversible and mediated through the inhibition of canonical Wnt signaling and the concomitant activation of the Wnt/PCP pathway (Figure 2B).

Increased levels of Wnt ligands are particularly protective against Aß-mediated cell toxicity. For example, exogenous Wnt3a prevents Aß neurotoxicity in cell lines and cultured hippocampal neurons (De Ferrari et al., 2003; Alvarez et al., 2004; Chacón et al., 2008). Aß-induced synaptic damage is also alleviated by Wnt5a in acute hippocampal slices, which prevents the dispersal of postsynaptic clusters and protects against defects in excitatory postsynaptic currents elicited by Aß (Cerpa et al., 2010). In summary, a number of studies strongly suggests that restoring or boosting Wnt signaling could protect cells and synapses from Aß toxicity and ameliorate AD pathology.

## Potential Side Effects of Boosting WNT Signaling

Given the role of Wnts in cancer, the challenge is how to develop treatments to boost Wnt signaling in neurodegenerative diseases without promoting cancer. Mutations in several Wnt signaling pathway components have been linked to colorectal cancer, in particular loss of function mutations in APC leading to high levels of active ß-catenin (Zhan et al., 2017). Also, transgenic models overexpressing Wnt1, and inducing high levels of ß-catenin activation, lead to mammary gland adenocarcinomas in mice (Tsukamoto et al., 1988). In addition, in several other cancers in which Wnt signaling plays a role, mutations in other oncogenic genes are required (White et al., 2012). For example, active ß-catenin levels are increased in a *Pten* knock-out hematopoietic stem cells induced T-lymphoblastic leukemia (T-ALL) mouse model (Guo et al., 2008). In this T-ALL model, the primary mutation is in the *Pten* gene, however, the ablation of one ß-catenin allele decreases the incidence and delays the appearance of the T-ALL (Guo et al., 2008). These studies suggest that high levels of the canonical Wnt signaling are required for cancer and that in many cases Wnt signaling activation is a secondary effect of other oncogenic mutations. In addition, different preclinical and clinical studies have tested GSK inhibitors as possible therapies for Alzheimer’s disease and for different types of cancer (Walz et al., 2017). Therefore, boosting Wnt signaling in a regulated manner could provide a viable approach for the treatment of neurodegenerative diseases without increasing the incidence of cancer.

## Conclusion

Compelling evidence supports the notion that Wnt signaling is deregulated in the aging brain and in AD (Figure 2B). Notably, the Wnt antagonist Dkk1 induces AD-related synaptic loss and cognitive impairment. Further mechanistic insights are required to elucidate how GSK3ß and ROCK activation lead to synapse loss in a synergic way. One could postulate that ROCK induces actin remodeling, which could lead to spine loss. However, the role of GSK3ß is less clear. Interestingly, the closely related protein Dkk3 is elevated in plasma and cerebrospinal fluid of AD patients, however, its role in the adult brain is still largely unexplored. In addition to Dkks, SFRPs represent another class of endogenous Wnt antagonists, which inhibit Wnt signaling by sequestering Wnt ligands. It is currently unknown whether these antagonists are similarly deregulated in the aging brain and in AD. Although two Wnt ligands are protective against Aß-induced synaptotoxicity in cultured neurons, their *in vivo* role has not been demonstrated. Moreover, we still lack a comprehensive view of the expression and activity of other Wnt ligands in the context of brain aging and AD. Further studies on key Wnt components such as Wnt secreted factors and Wnt receptors could provide more mechanistic insight into how deregulation of Wnt signaling contributes to cognitive decline during normal aging and in AD. Targeting components of the Wnt signaling pathway could open therapeutic avenues for boosting synaptic resilience and enhancing cognition in elderly people and people living with AD.

## Author Contributions

All authors contributed the same in the process of reading the literature and writing the manuscript. Figures were prepared by EP.

## Conflict of Interest Statement

The authors declare that the research was conducted in the absence of any commercial or financial relationships that could be construed as a potential conflict of interest.
